# Modulation of Connexin-36 Gap Junction Channels by Intracellular pH and Magnesium Ions

**DOI:** 10.3389/fphys.2018.00362

**Published:** 2018-04-12

**Authors:** Lina Rimkute, Tadas Kraujalis, Mindaugas Snipas, Nicolas Palacios-Prado, Vaidas Jotautis, Vytenis A. Skeberdis, Feliksas F. Bukauskas

**Affiliations:** ^1^Institute of Cardiology, Lithuanian University of Health Sciences, Kaunas, Lithuania; ^2^Department of Applied Informatics, Kaunas University of Technology, Kaunas, Lithuania; ^3^Department of Mathematical Modelling, Kaunas University of Technology, Kaunas, Lithuania; ^4^Centro Interdisciplinario de Neurociencias de Valparaíso, Universidad de Valparaíso, Valparaíso, Chile; ^5^Department of Physiology, Pontificia Universidad Católica de Chile, Santiago, Chile

**Keywords:** connexin-36, gap junctions, intracellular pH and Mg^2+^, mutants, cell culture

## Abstract

Connexin-36 (Cx36) protein forms gap junction (GJ) channels in pancreatic beta cells and is also the main Cx isoform forming electrical synapses in the adult mammalian brain. Cx36 GJs can be regulated by intracellular pH (pH_i_) and cytosolic magnesium ion concentration ([Mg^2+^]_i_), which can vary significantly under various physiological and pathological conditions. However, the combined effect and relationship of these two factors over Cx36-dependent coupling have not been previously studied in detail. Our experimental results in HeLa cells expressing Cx36 show that changes in both pH_i_ and [Mg^2+^]_i_ affect junctional conductance (g_j_) in an interdependent manner; in other words, intracellular acidification cause increase or decay in g_j_ depending on whether [Mg^2+^]_i_ is high or low, respectively, and intracellular alkalization cause reduction in g_j_ independently of [Mg^2+^]_i_. Our experimental and modelling data support the hypothesis that Cx36 GJ channels contain two separate gating mechanisms, and both are differentially sensitive to changes in pH_i_ and [Mg^2+^]_i_. Using recombinant Cx36 we found that two glutamate residues in the N-terminus could be partly responsible for the observed interrelated effect of pH_i_ and [Mg^2+^]_i_. Mutation of glutamate at position 8 attenuated the stimulatory effect of intracellular acidification at high [Mg^2+^]_i_, while mutation at position 12 and double mutation at both positions reversed stimulatory effect to inhibition. Moreover, Cx36^*^E8Q lost the initial increase of g_j_ at low [Mg^2+^]_i_ and double mutation lost the sensitivity to high [Mg^2+^]_i_. These results suggest that E8 and E12 are involved in regulation of Cx36 GJ channels by Mg^2+^ and H^+^ ions.

## Introduction

Cell-to-cell coupling through gap junction (GJ) channels is essential for intercellular communication in most cell types. GJ channels serve as an intercellular pathway for ions, small metabolites such as IP_3_ and cAMP (Niessen et al., 2000; Bedner et al., 2006), and larger molecules such as small interfering RNAs (Valiunas et al., 2005; Antanaviciute et al., 2014) and peptides (Neijssen et al., 2005). Electrotonic coupling through the GJs ensures propagation of action potentials between cardiomyocytes (Rohr, 2004), synchronization of neuronal activity in various brain regions (Bennett and Zukin, 2004) and is an important component of retinal circuitry (Völgyi et al., 2013). GJs play an important role in non-excitable tissue as well, since intercellular cell signalling via GJs may orchestrate proliferation (Vance and Wiley, 1999; Murray et al., 2009) and apoptosis (Kameritsch et al., 2013; Akopian et al., 2014).

GJ channels consist of two apposed hemichannels from contiguous cells. In vertebrates, each hemichannel is formed by six protein subunits of the connexin (Cx) family. Structural studies have revealed that Cxs comprise four transmembrane domains (M1-M4), two extracellular loops (E1 and E2), one cytoplasmic loop (CL), and cytoplasmic N- and C-termini (NT and CT). It is well-established that GJs formed of Cxs can be regulated by transjunctional voltage (V_j_) (Harris et al., 1981; Bukauskas and Verselis, 2004) or cytosolic conditions, such as intracellular pH (pH_i_) (Trexler et al., 1999; Palacios-Prado et al., 2010) or divalent cations (Noma and Tsuboi, 1987; Peracchia, 2004; Matsuda et al., 2010; Palacios-Prado et al., 2013). Conductance of GJs could be regulated by chemical uncouplers such as polyamines (Shore et al., 2001; Musa and Veenstra, 2003), alkanols (Weingart and Bukauskas, 1998), fenamates (Harks et al., 2001), antimalarial drugs (Srinivas et al., 2001; Cruikshank et al., 2004), and others. Cxs can also be affected by post-translational phosphorylation (Lampe and Lau, 2004; Moreno, 2005).

In humans, 21 different Cx isoforms have been identified (Söhl and Willecke, 2004). These isoforms are differentially expressed in various tissues and exhibit different biophysical and biochemical properties. Among the Cx family, Cx36 is mainly expressed in the adult mammalian central nervous system, where it forms electrical synapses. It has been shown that Cx36-containing electrical synapses play an important role in facilitating synchronous or phase-locked activity of neuronal networks, which underlie a variety of cognitive processes (Bennett and Zukin, 2004; Connors and Long, 2004; Hormuzdi et al., 2004; Saraga et al., 2006; Bissiere et al., 2011). Cx36 also forms GJs between pancreatic beta cells, where it plays an important role in insulin secretion and glycaemic control (Farnsworth and Benninger, 2014).

As compared with other Cx isoforms, Cx36 GJ channels have some distinct biophysical and regulatory properties. For example, Cx36 exhibits a very low unitary conductance and low sensitivity to transjunctional voltage (Srinivas et al., 1999; Teubner et al., 2000). Its regulation by pH_i_ and free cytosolic Mg^2+^ ion concentration ([Mg^2+^]_i_) also has some distinctive characteristics. Unlike other Cx isoforms, junctional conductance (g_j_) of Cx36 GJs can be upregulated under low pH_i_ (González-Nieto et al., 2008) and low [Mg^2+^]_i_ (Palacios-Prado et al., 2013). In addition, [Mg^2+^]_i_ can change Cx36 sensitivity to transjunctional voltage. Studies with recombinant Cx36 revealed that Mg^2+^-dependent regulation of g_j_ may be explained via electrostatic interaction with a binding site located in the channel pore (Palacios-Prado et al., 2014). Preliminary results showed that the effect of [Mg^2+^]_i_ and pH_i_ on Cx36 GJs might be interrelated (Palacios-Prado et al., 2011). This raised the hypothesis that Mg^2+^ and H^+^ ions may interact on the same binding sites, as was shown for TRPM7 ion channels (Jiang et al., 2005). However, the effect on g_j_ of Cx36 GJs produced by combined changes in [Mg^2+^]_i_ and pH_i_ has not been studied in detail.

Both pH_i_ and [Mg^2+^]_i_ are known to play an important role in normal and various pathological conditions. For example, increased neural activity may cause a shift in pH_i_ of 0.2–0.4 units (Chesler and Kraig, 1989; Chesler and Kaila, 1992) under physiological conditions, which may subsequently modulate electrical synapses. In addition, the depletion of ATP during brain ischemia (Sato et al., 1984) may cause an increase of [Mg^2+^]_i_ (Henrich and Buckler, 2008). The connection between Mg^2+^ deficiency and formation of epileptic seizures is well-established (Randall et al., 1959; Hanna, 1961; Nuytten et al., 1991), and the role of electrical synapses and Cx36 in epilepsy is an important topic of research (Gajda et al., 2005; Volman et al., 2011; Kohmann et al., 2016; Wu et al., 2017). Furthermore, brain [Mg^2+^]_i_ is decreased in patients with Alzheimer's and Parkinson's disease (Durlach, 1990; Barbiroli et al., 1999), while patients with schizophrenia and traumatic brain injury show increased brain [Mg^2+^]_i_ (Hinsberger et al., 1997). In the pancreas, low pH_i_ plays an important role in glucose-induced insulin release, while low [Mg^2+^]_i_ is associated with decreased insulin secretion (Ishizuka et al., 1994) and pancreatitis (Papazachariou et al., 2000). Thus, understanding the interaction of pH_i_ and [Mg^2+^]_i_ and their combined effects on gap junctional communication (GJC) could reveal new modulatory mechanisms of GJs in physiology and pathology.

In this study, we examined the combined effect of [Mg^2+^]_i_ and pH_i_ on g_j_ between cells expressing Cx36-EGFP. Our data revealed that after g_j_ was reduced by high [Mg^2+^]_i_, it could be recovered by intracellular acidification with sodium acetate (CH_3_COONa). In contrast, after g_j_ was elevated by low [Mg^2+^]_i_, both alkalization or acidification with ammonium chloride (NH_4_Cl) or CH_3_COONa, respectively, induced a reduction of g_j_. To consider the most appropriate amino acids which could be involved in Cx36 GJ channel regulation by H^+^, we used homology modelling to generate a three-dimensional structure of Cx36 using the crystal structure of Cx26 (Maeda et al., 2009) as a template, which allowed us to estimate the pK_a_ of all ionizable amino acid side chains. The calculations showed that two glutamates (E8 and E12) exhibited pK_a_ values which were closest to physiological pH. Thus, we performed experiments with three Cx36 mutants, Cx36^*^E8Q-EGFP, Cx36^*^E12Q-EGFP and Cx36^*^E8Q-E12Q-EGFP, in which negatively charged glutamates were substituted to uncharged glutamines. Experimental results showed that acidification-induced g_j_ increase at high [Mg^2+^]_i_ in Cx36-EGFP was abolished in Cx36^*^E8Q-EGFP, while Cx36^*^E12Q-EGFP exhibited a small g_j_ decrease under the same conditions. The most prominent decrease of g_j_ at high [Mg^2+^]_i_ during acidification was observed in double mutant Cx36^*^E8Q-E12Q-EGFP. Therefore, these amino acids could be involved in modulatory mechanisms of Cx36 GJ channels by both, [Mg^2+^]_i_ and pH_i_.

## Materials and methods

### Structural modelling

Cx36 homology modelling was carried out with MODELLER (version 9.10) (Webb and Sali, 2014), using a Cx26 crystal structure (Maeda et al., 2009) as a template. The prediction of pK_a_ values of ionizable groups in Cx36 was based on the 3D structure and was performed with PROPKA (version 3.0) (Olsson et al., 2011; Søndergaard et al., 2011).

### Cell and culture conditions

Electrophysiological measurements were performed using HeLa (human cervix carcinoma cells, ATCC CCL2) cells transfected with wild type mouse Cx36 fused with enhanced green fluorescent protein (EGFP). Cells were grown in Dulbecco's Modified Eagle's Medium (DMEM) supplemented with 10% fetal calf serum. Cells were maintained in a 5% CO_2_ incubator in a moist atmosphere at 37 ^O^C. Media and culture reagents were obtained from Sigma-Aldrich, Germany. Single point mutations, Cx36^*^E8Q-EGFP and Cx36^*^E12Q-EGFP, and double point mutation, Cx36^*^E8Q-E12Q-EGFP were generated using the QuikChange Multi Site-directed mutagenesis kit (Agilent, USA). Mutants were subcloned into *pIRESPuro2* vector (Clontech, USA). Transfection procedures were performed using Lipofectamine 2000 (Life technologies, USA) following the manufacturer's protocol.

### Electrophysiological measurements

For simultaneous electrophysiological and fluorescence recordings, cells grown on glass coverslips were transferred to an experimental chamber mounted on the stage of an inverted microscope Olympus IX71 (Olympus, Japan) with a constant flow-through perfusion. The g_j_ was measured in selected cell pairs by using a dual whole-cell patch clamp. Cell-1 and cell-2 of a cell pair were voltage clamped independently with separate patch clamp amplifiers EPC-8 (HEKA Elektronik, Germany) at the same holding potential, V_1_ = V_2_. Voltages and currents were acquired and analysed using an analog-to-digital converter (National Instruments, Austin, TX) and custom-made software. By stepping the voltage in cell-1 (ΔV_1_) and keeping the other constant, junctional current was measured as the change in current in the unstepped cell-2, I_j_ = –ΔI_2_. Thus, g_j_ was obtained from the ratio -I_j_/ΔV_1_, where ΔV_1_ is equal to V_j_ and the negative sign indicating that I_j_ measured in cell-2 is oppositely oriented to the one measured in cell-1. To minimize the effect of series resistance on measurements of g_j_, we maintained recording pipette resistance below 3 MΩ. Patch pipettes were pulled from glass capillary tubes with filaments using a P-97 micropipette puller (Sutter Instrument Co., US).

Cells were perfused with modified Krebs-Ringer (MKR) solution containing (in mM): 140 NaCl, 4 KCl, 2 CaCl_2_, 1 MgCl_2_, 2 CsCl, 1 BaCl_2_, 5 HEPES, 5 glucose, 2 pyruvate, pH 7.4. Changes of pH_i_ were achieved by using NH_4_Cl and CH_3_COONa to alkalize and acidify, respectively, the intracellular milieu without a change in extracellular pH (pH_o_). Recording pipettes were filled with solution containing (in mM): 130 CsCl, 10 NaAsp, 0.26 CaCl_2_, 5 HEPES, 2 BAPTA, 1 MgCl_2_, pH 7.3. To investigate the effect of [Mg^2+^]_i_ we used pipette solutions containing 0.01, 1 or 5 mM of MgCl_2_. Differences in osmolarity were compensated with the appropriate concentration of CsCl.

To prepare solutions for intracellular acidification and alkalization during experiments, we used modified Ringer's solution in which NaCl was exchanged for equal concentration of CH_3_COONa or NH_4_Cl. All extracellular solutions were adjusted to pH = 7.4. To reduce pH_i_ to 6.5 and 6.0, we used physiological solution containing 20 and 100 mM of CH_3_COONa, respectively, and to increase pH_i_ to 7.6, 7.9, and 8.2, we added to the physiological solution 1, 3, and 10 mM of NH_4_Cl, respectively (Table 1).

**Table 1:** pH_i_ values measured with BCECF during acidification with CH_3_COONa and alkalization with NH_4_Cl.

**Concentration**	**100 mM CH_3_COONa**	**20 mM CH_3_COONa**	**Control**	**1 mM NH_4_Cl**	**3 mM NH_4_Cl**	**10 mM NH_4_Cl**
pH_i_	6.02 ± 0.05	6.53 ± 0.05	7.27 ± 0.07	7.64 ± 0.02	7.87 ± 0.03	8.15 ± 0.01

### Fluorescence imaging studies

Fluorescence signals were acquired using UltraVIEW (PerkinElmerLifeSciences, Boston, MA, US) software. An excitation filter of 470 nm, and emission filter of 540 nm were used to identify the cell pairs expressing Cx36-EGFP and its mutants. For pH_i_ measurements we used 4 μM BCECF (Invitrogen, USA), which was introduced into the cells through the patch pipettes in a whole-cell voltage clamp mode. The dye was alternately excited with 436 and 500 nm wavelengths, and the emitted light was filtered at 540 nm. The ratios of emitted light collected at excitation wavelengths of 436 and 500 nm (background subtracted) were converted to pH_i_ values based on a calibration curve. The latter was obtained by applying the solutions of different pH (6.0, 6.5, 7.0, 7.5, 8.0, and 8.5) and using ionophore nigericin (20 μM) in the presence of potassium (140 mM) to equilibrate the pH_i_ with an extracellular medium of different pH (Thomas et al., 1979). The pH_i_ values at different concentrations of CH_3_COONa and NH_4_Cl are presented in Table 1. To prevent dye bleaching, imaging was performed in time-lapse mode by exposing every 15 s to a low-intensity excitation light for 500 ms.

### Statistical analysis

Experimental data are reported as the representative result or as mean of at least four independent experiments ± standard error (SEM). Statistical analyses were performed using unpaired Student's *t*-test. Differences were considered statistically significant at *p* < 0.05.

## Results

### The effect of H^+^ and intracellular Mg^2+^ on Cx36 GJ channel function

**Figure 1:**
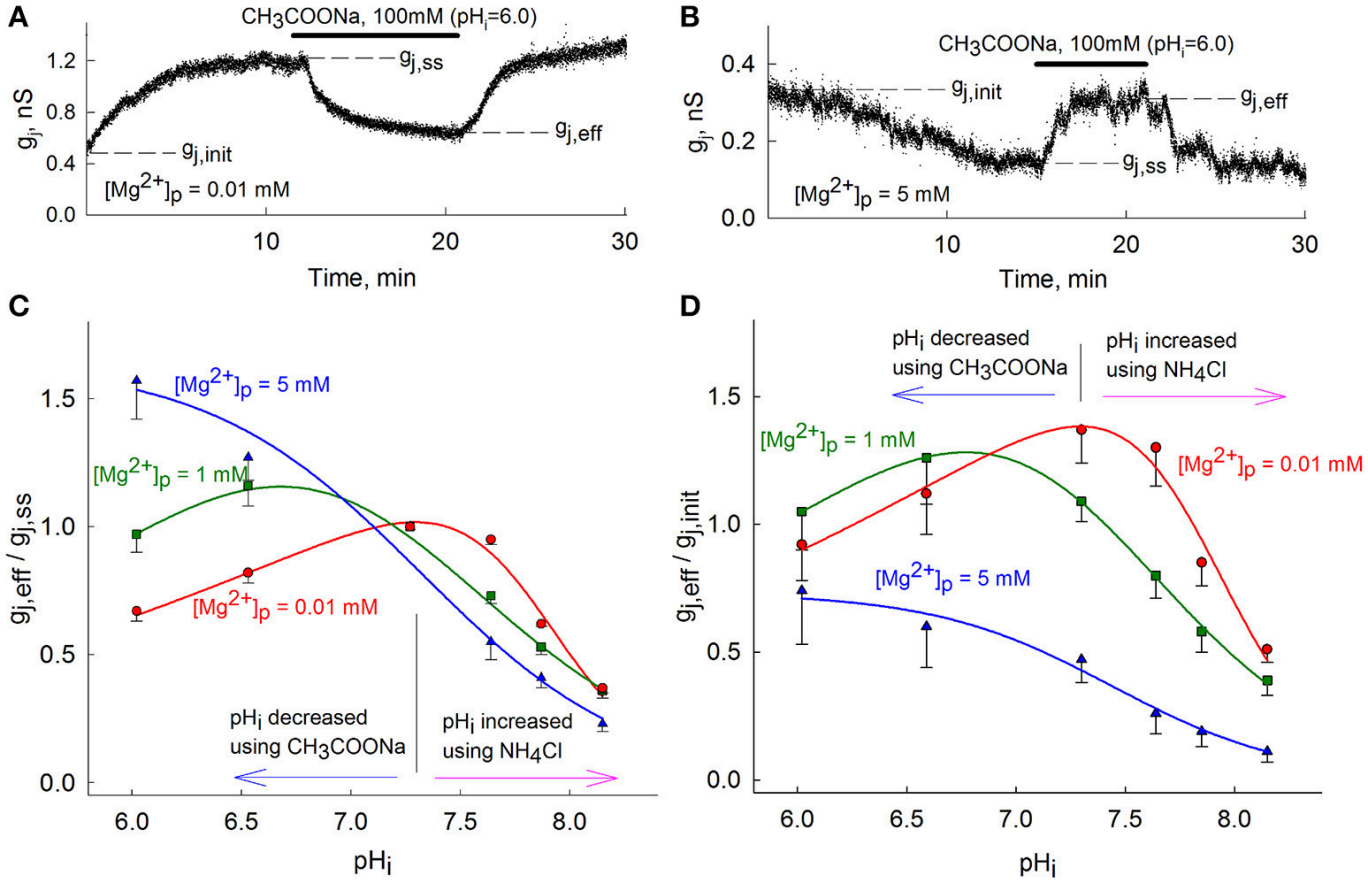
Modulation of g_j_-pH_i_ by [Mg^2+^]_p._ **(A,B)** g_j_ measurements over time using low **(A)** or high **(B)** [Mg^2+^]_p_ followed by CH_3_COONa-induced acidification to pH_i_ = 6.0; **(C)** The (g_j, eff_/g_j, ss_)-pH_i_ dependence of Cx36-EGFP GJs at different [Mg^2+^]_p_. Data are normalized to g_j, ss_ at each different [Mg^2+^]_p_ before changes in pH_i_. **(D)** The same as in **(C)**, but normalized to g_j, init_. To avoid overlap, only lower ranges of standard error are presented in **(C,D)**. Vertical black lines in **(C,D)** are located at pH_i_ = 7.3.

Preliminary data suggested that [Mg^2+^]_i_ can substantially modulate g_j_-pH_i_ dependence of Cx36-EGFP GJs (Palacios-Prado et al., 2011). To study the relation of [Mg^2+^]_i_ and pH_i_, and their combined effect on g_j_ more systematically, we examined g_j_-pH_i_ dependence in HeLa cells expressing Cx36-EGFP cells at different concentrations of Mg^2+^ (0.01, 1, and 5 mM) in pipette solutions ([Mg^2+^]_p_). In these experiments, the initial g_j_ (g_j, init_) at control pH_i_ = 7.3 was registered immediately after patch opening. Typically, g_j_ increased or decreased at low or high [Mg^2+^]_i_, respectively, until it reached a steady-state (g_j, ss_) (Figures 1A,B). Then, we decreased or increased pH_i_ by applying different concentrations of CH_3_COONa or NH_4_Cl, respectively, and measured g_j_ (g_j, eff_) before washing out the applied substance. To prepare these solutions, we used modified Ringer's solution in which NaCl was exchanged for equal concentration of CH_3_COONa or NH_4_Cl to maintain osmolarity. Extracellular pH (pH_o_) remained unchanged during the experiments. Solution and pH_i_ measurement protocols are presented in the Methods section. The control experiments in non-transfected HeLa cells showed low conductance in very rare cases (1 in 20), which could be attributed to activity of endogenous connexins in these cells (data not shown).

Figure 1C shows g_j, eff_/g_j, ss_ dependence on pH_i_ at different [Mg^2+^]_p_. The mean values of (g_j, eff_/g_j, ss_)-pH_i_ dependence at 0.01 and 1 mM [Mg^2+^]_p_ were fitted to an equation describing biphasic effects (Swietach et al., 2007) and a sigmoid function was used to fit data at 5 mM [Mg^2+^]_p_. Alkalization to pH_i_ = 8.2 caused a ~70% decay of g_j, eff_/g_j, ss_ almost independently of [Mg^2+^]_p_. However, changes of g_j_ during acidification highly varied depending on [Mg^2+^]_p_. At [Mg^2+^]_p_ = 0.01 mM, acidification to pH_i_ = 6.0 decreased g_j, eff_/g_j, ss_ to 0.67 ± 0.04 (*n* = 4). At [Mg^2+^]_p_ = 1 mM, acidification to pH_i_ = 6.6 first induced an increase of g_j, eff_/g_j, ss_ to 1.16 ± 0.08 (*n* = 8), while further acidification to pH_i_ = 6.0 returned g_j, eff_/g_j, ss_ to 0.97±0.07 (n = 12). At [Mg^2+^]_p_ = 5 mM, acidification to pH_i_ = 6.0 increased g_j, eff_/g_j, ss_ to 1.57 ± 0.15 (*n* = 4). Figure 1D shows the ratios g_j, eff_/g_j, init_ at different [Mg^2+^]_p_, to represent the effect of [Mg^2+^]_i_ on g_j_ and the consequent changes by altering pH_i_; at pH_i_ = 7.3 this ratio coincides with g_j, ss_/g_j, init_. From Figure 1D it can be seen that peaks of (g_j, eff_/g_j, init_)-pH_i_ dependencies shift toward the acidic side as the value of [Mg^2+^]_p_ increases. Therefore, g_j, eff_/g_j, init_ reaches a maximum value at pH_i_ range between 7.1–7.5, 6.6–6.9 and below 6.0 for [Mg^2+^]_p_ of 0.01, 1, and 5 mM, respectively. These experimental results clearly show that pH_i_ and [Mg^2+^]_i_ regulation of Cx36 is interrelated, particularly at lower pH_i_ values.

### The role of glutamates in Cx36 GJ sensitivity to pH_i_ and [Mg^2+^]_i_

Some studies have shown that gating polarity and single channel conductance of GJ channels are determined by charged amino acids of the NT domain (Verselis et al., 1994; Musa et al., 2004; Tong and Ebihara, 2006), which forms the vestibule of the channel. We hypothesized that negatively charged amino acids of the NT domain could play an important role in Mg^2+^ ions' interaction with Cx36 protein as they form the path for Mg^2+^ ions to enter the channel and could be involved in their binding. In order to determine the amino acids which also could be sensitive to pH_i_, the x-ray crystal structure of Cx26 (Maeda et al., 2009) was used to generate the homology model of Cx36 with MODELLER software, and pK_a_ values were evaluated using PROPKA software for all ionizable amino acid side chains depending on their environment. Results showed that glutamates at positions 8 and 12 (E8 and E12) have pK_a_ values equal to 6.5 and 7.2, respectively. These pK_a_ values were the closest to physiological pH_i_ among other amino acids in the NT domain of Cx36, thus indicating that carboxyl groups at side chains of E8 and E12 may be sensitive to protonation and deprotonation at physiological pH_i_ values. Based on these predictions, E8 and E12 were chosen for point mutations. As substitutes for glutamates, we chose glutamines (E8Q or E12Q, respectively), which are neutral but polar. Therefore, the substitution of glutamate by glutamine neutralizes the negative charge and abolishes channel protonation, which possibly affects the dynamic behavior of a gate that is pH-sensitive and/or Mg^2+^-sensitive. Moreover, double mutation, where both E8 and E12 were substituted by glutamines, was also analysed.

**Figure 2:**
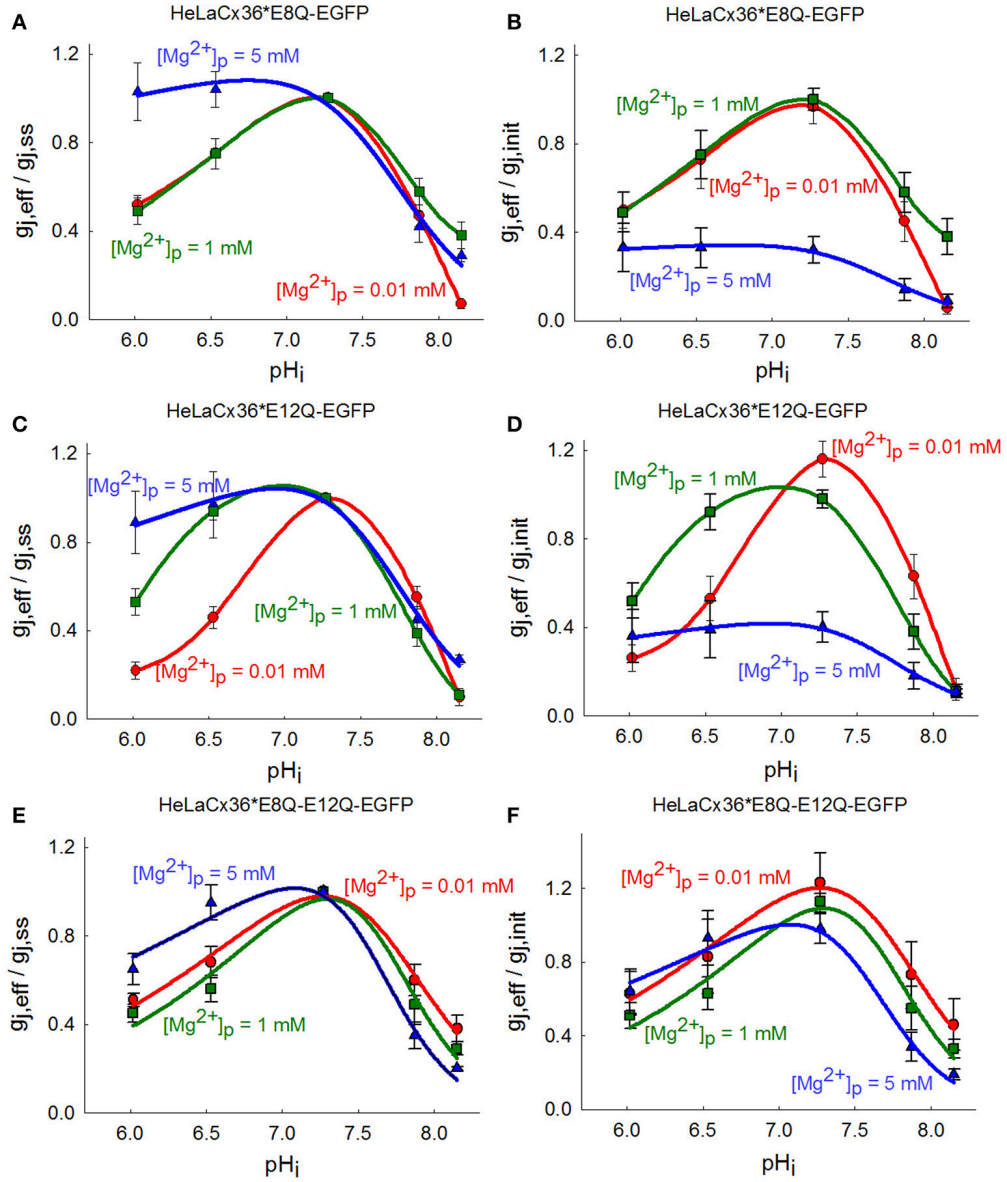
Influence of single and double E8Q and E12Q mutations on (g_j, eff_/g_j, ss_)-pH_i_ dependence modulated by [Mg^2+^]_p_. **(A)** For Cx36*E8Q-EGFP the effect of acidification is modulated by high [Mg^2+^]_p_, while alkalization—by low [Mg^2+^]_p_. **(B)** The increase of [Mg^2+^]_p_ shifts peaks of Cx36*E8Q-EGFP (g_j, eff_/g_j, init_)-pH_i_ curves to the left with the maximal g_j, eff_/g_j, init_ values at pH_i_ ~6.9–7.4 for 0.01 and 1 mM [Mg^2+^]_p_, and at pH_i_ ~6.7–7.2 for 5 mM [Mg^2+^]_p_. **(C)** The decrease of g_j, eff_/g_j, ss_ of Cx36*E12Q-EGFP during acidification depends on [Mg^2+^]_p_. **(D)** The peaks of (g_j, eff_/g_j, init_)-pH_i_ dependencies shift to the left with the maximal g_j, eff_/g_j, init_ values at pH_i_ ~7.1–7.6 for 0.01 mM [Mg^2+^]_p_, and at pH_i_ ~6.7–7.3 for 1 and 5 mM [Mg^2+^]_p_. **(E)** Double mutation causes decrease of g_j, eff_/g_j, ss_ at high [Mg^2+^]_p_. **(F)** The peaks of (g_j, eff_/g_j, init_)-pH_i_ dependencies are the same at 0.01 and 1 mM [Mg^2+^]_p_, and only slightly shift to the left with the maximal g_j, eff_/g_j, init_ values at pH_i_ ~6.9–7.3 for 5 mM [Mg^2+^]_p_.

The experimental results showed that acidification to pH_i_ 6.0 and 6.5 decreased g_j, eff_/g_j, ss_ of Cx36^*^E8Q-EGFP approximately to the same level at 0.01 and 1 mM [Mg^2+^]_p_ (Figure 2A), and at [Mg^2+^]_p_ = 5 mM acidification to pH_i_ 6.0 and 6.5 resulted in a slight increase of g_j, eff_/g_j, ss_. During alkalization to pH_i_ = 8.2 g_j, eff_/g_j, ss_ decreased ~60–90% at all [Mg^2+^]_p_. Figure 2C shows that the uncoupling effect of acidification on Cx36^*^E12Q-EGFP GJ channels is highly dependent on [Mg^2+^]_p_. The strongest decrease of g_j, eff_/g_j, ss_ was reached at 0.01 mM [Mg^2+^]_p_, an ~50% decrease of g_j, eff_/g_j, ss_ was obtained at 1 mM [Mg^2+^]_p_, and a slight decrease to 0.89 ± 0.14 (*n* = 10) was observed at [Mg^2+^]_p_ = 5 mM. During alkalization to pH_i_ = 8.2 the g_j, eff_/g_j, ss_ decreased ~70–90% at all [Mg^2+^]_p._ Acidification to pH_i_ 6.0 and 6.5 decreased g_j, eff_/g_j, ss_ of double mutant ~50 and 40% at 0.01 and 1 mM [Mg^2+^]_p_, respectively, and ~35 and 5% at 5 mM [Mg^2+^]_p_, respectively (Figure 2E). The uncoupling of alkalization was dependent on [Mg^2+^]_p_ with strongest effect at 5 mM [Mg^2+^]_p_ for Cx36^*^E8Q-E12Q-EGFP. Figures 2B,D,F show that the shift of peaks of (g_j, eff_/g_j, init_)-pH_i_ dependencies for all mutants were less influenced by rising [Mg^2+^]_p_ than it is for Cx36-EGFP.

**Figure 3:**
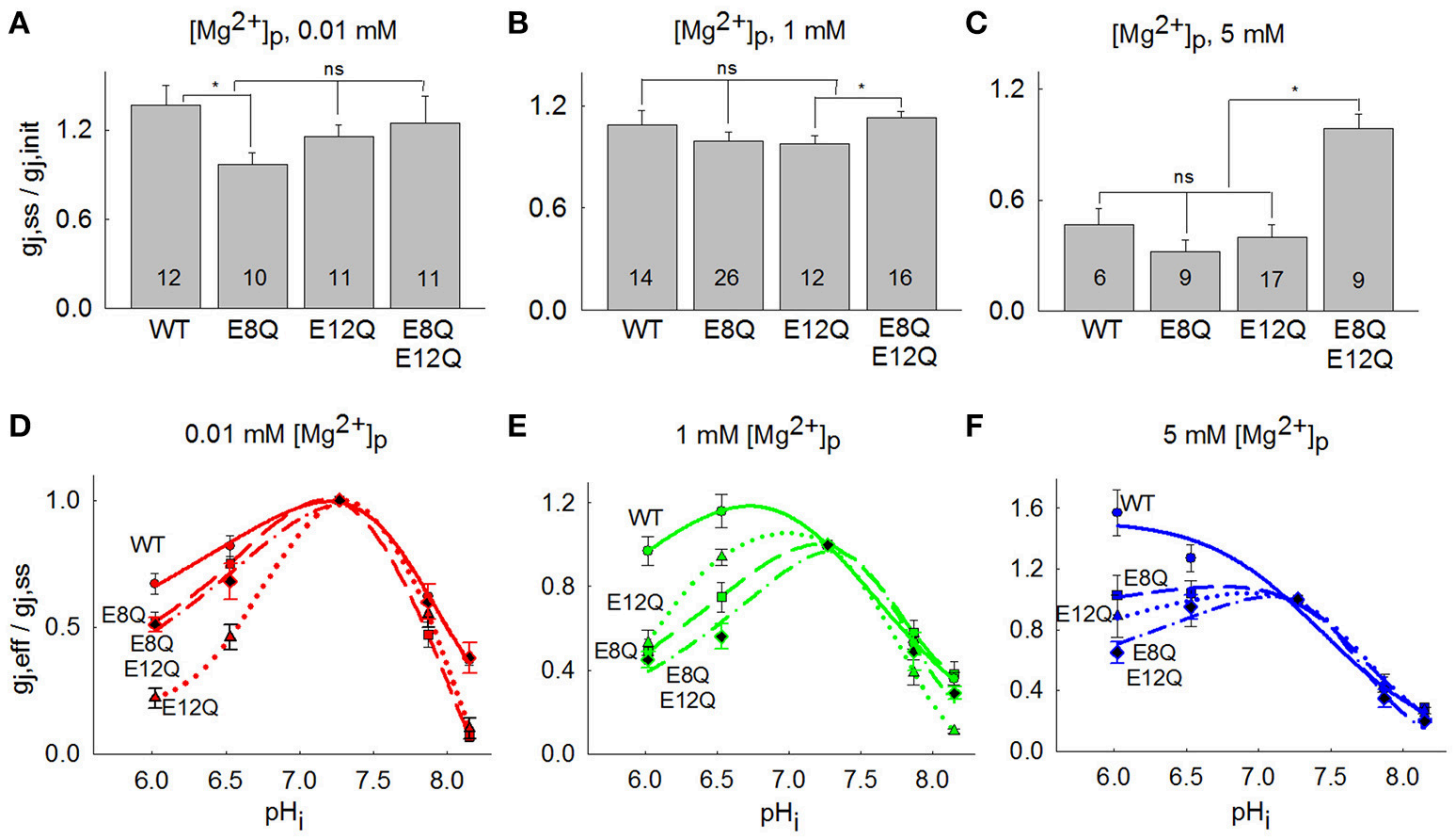
Comparison between g_j_ response of Cx36-EGFP, Cx36*E8Q-EGFP, Cx36*E12Q-EGFP and Cx36*E8Q-E12Q-EGFP to [Mg^2+^]_p_ and pH_i_. **(A)** The increase of g_j, ss_/g_j, init_ at 0.01 mM [Mg^2+^]_p_ was observed for Cx36-EGFP (1.37 ± 0.13), E12Q (1.16 ± 0.08), and E8Q-E12Q (1.23 ± 0.16), while a small decrease was obtained for E8Q (0.97 ± 0.08). **(B)** The g_j, ss_/g_j, init_ values at 1 mM [Mg^2+^]_p_ were 1.09±0.08, 1.00 ± 0.05, 0.98 ± 0.04, and 1.13 ± 0.04 for Cx36-EGFP, E8Q, E12Q, and E8Q-E12Q, respectively. **(C)** The decrease of g_j, ss_/g_j, init_ to 0.46 ± 0.09, 0.32 ± 0.06, and 0.40 ± 0.07 were obtained for Cx36-EGFP, E8Q, and E12Q, respectively, and for E8Q-E12Q mutation g_j, ss_/g_j, init_ was 0.98 ± 0.08. Number of performed experiments are indicated on the bars; *p < 0.05, ns, non-significant. **(D–F)** All mutations enhance the uncoupling effect of acidification at 0.01 and 1 mM [Mg^2+^]_p_, single E8Q mutation abolish the stimulating effect of acidification at 5 mM [Mg^2+^]_p_, while E12Q and double mutation E8Q-E12Q reverses it to a decrease in g_j, eff_/g_j,ss_.

The summarized changes in g_j, ss_/g_j, init_ of Cx36-EGFP, Cx36^*^E8Q-EGFP, Cx36^*^E12Q-EGFP and Cx36^*^E8Q-E12Q-EGFP are presented in Figures 3A–C, which shows that the effect of [Mg^2+^]_p_ on g_j, ss_/g_j, init_ for all mutants is comparable to Cx36-EGFP, with the exception of the E8Q mutation, which abolishes the increase of g_j_ at low [Mg^2+^]_p_ (Figure 3A), and the E8Q-E12Q mutation, which eliminates the decrease of g_j_ at high [Mg^2+^]_p_ (Figure 3C). The comparison of (g_j, eff_/g_j, ss_)-pH_i_ between three mutants and Cx36-EGFP is represented in Figures 3D–F. Acidification at low [Mg^2+^]_p_ causes the strongest uncoupling of cells transfected with Cx36^*^E12Q-EGFP (Figure 3D). Importantly, acidification did not cause any change of g_j, eff_/g_j, ss_ of Cx36^*^E8Q-EGFP and decreased g_j, eff_/g_j, ss_ of Cx36^*^E12Q-EGFP and Cx36^*^E8Q-E12Q-EGFP, while g_j, eff_/g_j, ss_ of Cx36-EGFP is stimulated at high [Mg^2+^]_p_ (Figure 3F). All mutants show an increased sensitivity to the uncoupling effect of acidification at 1 mM [Mg^2+^]_p_ as compared with Cx36-EGFP (Figure 3E). Changes in sensitivity to alkalization is less visible, however alkalization causes a stronger decrease of Cx36^*^E12Q-EGFP g_j_ than Cx36-EGFP at low and normal [Mg^2+^]_p_. Sensitivity of Cx36^*^E8Q-EGFP to alkalization is similar to Cx36-EGFP at normal [Mg^2+^]_p_ and is close to Cx36^*^E12Q-EGFP at low [Mg^2+^]_p_. No significant changes between Cx36-EGFP and all mutants were observed in uncoupling by alkalization at high [Mg^2+^]_p_.

In summary, the effect of [Mg^2+^]_p_ on g_j, ss_/g_j, init_ of Cx36^*^E8Q-EGFP and Cx36^*^E12Q-EGFP remained comparable to that of Cx36-EGFP with the exception that E8Q abolished the increase of g_j, ss_/g_j, init_ at low [Mg^2+^]_p_ and E8Q-E12Q lost the sensitivity to high [Mg^2+^]_p_. Moreover, E8Q diminished the stimulating effect of acidification, while E12Q as well as E8Q-E12Q caused the decrease of g_j, eff_/g_j, ss_ instead of stimulation, which was shown for Cx36-EGFP at high [Mg^2+^]_p_. These results show that mutations disturb the interrelated effect of [Mg^2+^]_i_ and pH_i_ on regulation of the Cx36 GJ channel.

### Mathematical model of Cx36 regulation by PH_i_ and [Mg^2+^]_i_

Data showing that uncoupling can be observed by both increased and decreased [H^+^]_i_ suggest that Cx36 may contain pH_i_ sensitive domains, one that leads to g_j_ stimulation and another one that leads to g_j_ inhibition. This hypothesis was previously raised in González-Nieto et al. (2008), where the authors postulated the existence of alkalic and acidic gates in Cx36 GJ channels. We applied this idea to model the mean values of g_j, eff_/g_j, ss_ at different [Mg^2+^]_i_ and pH_i_. We assumed that g_j_ can be regulated by alkalic and acidic gating mechanisms, which are described by open channel probabilities in response to alkalization and acidification, *p*_*alk*_ and *p*_*acid*_, respectively. The probabilities *p*_*alk*_ and *p*_*acid*_ were described by sigmoid function as follows:

(1) $$\begin{array}{r} p_{alk\;or\;acid}=\;\frac{e^{A\left({pH}_{i}\;-\;{pH}_{1/2}\right)}}{1+\;e^{A\left({pH}_{i}\;-\;{pH}_{1/2}\right)}} \end{array}$$

Here, parameter *A* describes the steepness of the sigmoidal curve, and pH_1/2_ denotes the pH_i_ level at which open channel probability is equal to 0.5. Then, overall g_j_ can be estimated as a product of maximum junctional conductance (*g*_*max*_) and open channel probabilities determined by both acidic and alkalic sensing domains:

(2) $$\begin{array}{r} g_{j}=\;g_{\max}\times \;p_{alk}\times \;p_{acid} \end{array}$$

**Figure 4:**
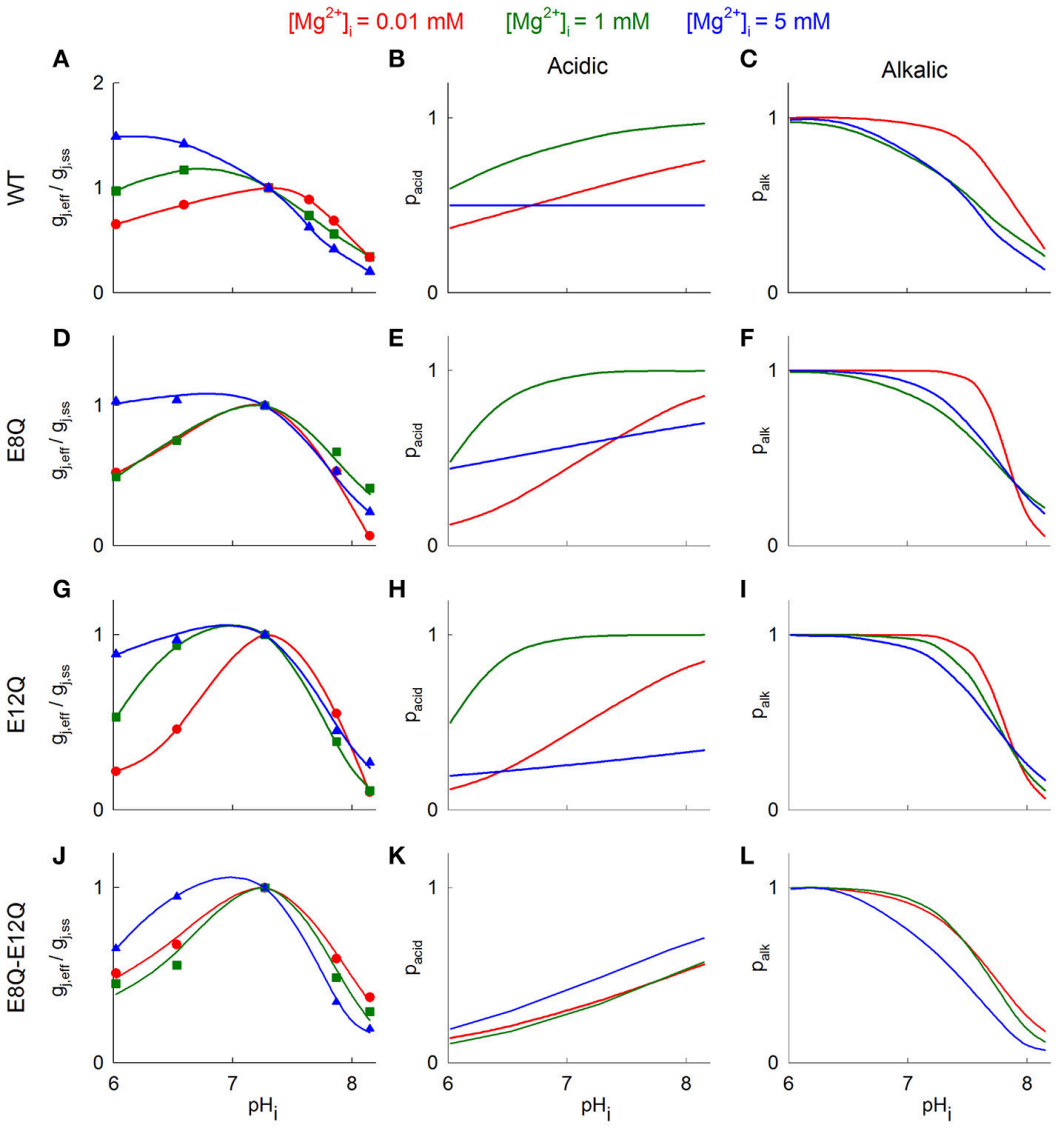
Mathematical model of g_j_ changes in response to pH_i_ under different [Mg^2+^]_i_. **(A)** Hypothetical g_j_-pH_i_ curves fitted to experimental data. Blue triangles denote g_j_ values at [Mg^2+^]_p_ = 5 mM, green squares—at [Mg^2+^]_p_ = 1 mM, and red circles - at [Mg^2+^]_p_ = 0.01 mM. The theoretical g_j_-pH_i_ plots were obtained from the Equation (2). **(B,C)** shows theoretical open channel probabilities in response to acidic gating **(B)** and alkalic gating **(C)**, which were estimated from (1). Red, green and blue lines denote g_j_-pH_i_ dependence at [Mg^2+^]_i_ = 0.01, 1, and 5 mM, respectively. **(D–L)** show the same results as **(A–C)** with Cx36 mutants.

Figure 4 illustrates fitted g_j_ curves of *p*_*acid*_ and *p*_*alk*_ under different [Mg^2+^]_i_ to reproduce experimentally observed g_j_ values. The model parameters at different levels of [Mg^2+^]_i_ are presented in Table 2. Model fitting shows that p_acid_ of Cx36-EGFP does not depend on pH_i_ at high [Mg^2+^]_i_ and increases with pH_i_ rising at lower [Mg^2+^]_i_ (Figure 4B). In contrast, p_alk_ decreased at higher pH_i_ and this reduction does not depend on [Mg^2+^]_i_ (Figure 4C).

**Table 2:** Parameters of model of open channel probabilities determined by acidic and alkalic sensing domains at different levels of [Mg^2+^]_i_.

	**[Mg^2+^]_i_, mM**	**A_acid_**	**pH_1/2, acid_**	**A_alk_**	**pH_1/2, alk_**	**g_max_, nS**
Cx36-EGFP	0.01	0.77	6.70	4.23	7.90	2.42
	1	1.42	5.75	2.34	7.59	1.80
	5	0.00	5.39	3.00	7.53	1.39
Cx36*E8Q-EGFP	0.01	1.65	6.16	9.05	7.84	1.31
	1	1.43	6.77	2.83	7.70	1.91
	5	0.32	5.03	3.64	7.74	0.55
Cx36*E12Q-EGFP	0.01	1.77	7.15	7.71	7.81	2.13
	1	3.53	6.04	5.15	7.75	1.08
	5	0.51	6.49	3.59	7.71	0.80
Cx36*E8Q-E12Q-EGFP	0.01	0.97	7.89	3.44	7.71	4.22
	1	1.12	7.88	4.16	7.67	3.99
	5	1.10	7.32	3.41	7.40	3.32

The p_acid_ of all mutants gained the dependence on pH_i_ at high [Mg^2+^]_i_ (Figures 4E,H,**K** blue lines) with most prominent difference for Cx36^*^E8Q-E12Q-EGFP. In addition, overall p_acid_ values were significantly reduced for Cx36^*^E12Q-EGFP at [Mg^2+^]_i_ = 5 mM and for double mutant these values were reduced at 1 and 5 mM [Mg^2+^]_i_. The dependence of p_alk_ on pH_i_ remains comparable to that of Cx36-EGFP and does not significantly change at all [Mg^2+^]_i_ for all three mutants (Figures 4C,F,I,L).

## Discussion

The distinct effects of pH_i_ and divalent ion concentrations on the conductance of GJ channels and hemichannels have been known for a long time and have been reported in many studies. The effect of pH_i_ on electrical coupling was demonstrated even before the sequencing of the Cx gene family (Rose and Loewenstein, 1975; Turin and Warner, 1977; Giaume and Korn, 1982). Most Cx isoforms have been found to be modulated by pH_i_ (Hermans et al., 1995; Wang and Peracchia, 1996; Palacios-Prado et al., 2010). Intracellular divalent cations have also been shown to modulate g_j_ of GJ channels (Peracchia, 1990; Matsuda et al., 2010; Harris and Contreras, 2014). The interaction between pH_i_ and [Ca^2+^]_i_ and their consequent effect on g_j_ have been previously studied (Peracchia, 2004). Nonetheless, we recently found that changes in [Mg^2+^]_i_ strongly affect g_j_ in Cx36 (Palacios-Prado et al., 2013); (Palacios-Prado et al., 2014), which encouraged us to study the possible interaction between pH_i_ and [Mg^2+^]_i_ and their interrelated effect on Cx36-dependent coupling. In this study, we present data demonstrating that pH_i_ and [Mg^2+^]_i_ have an interrelated effect on the function of Cx36 GJ channels, and showed that two glutamate residues in NT domain are involved in this modulation.

### Free intracellular H^+^ and Mg^2+^ ions interact with channel residues

Mg^2+^ and H^+^ are known to participate in a variety of physiological processes and could exert differential effects on many cellular targets. There are many different ways by which both pH_i_ and [Mg^2+^]_i_ could affect conductance of GJ channels and hemichannels. Therefore, it is not surprising that a variety of possible mechanisms have been proposed to account for their effect. For example, in Bevans and Harris (1999) and Tao and Harris (2004) it was proposed that H^+^ ions affect gating of Cx26 GJ channels and hemichannels via protonation of taurine, which can inhibit GJ channel activity. In addition, Peracchia (2004) suggested that the uncoupling effect of pH_i_ in most cases could be explained through an increase in [Ca^2+^]_i_. However, some studies have also shown that H^+^ could affect conductance of GJ channels and hemichannels via direct protonation of Cx residues. For example, Spray et al. (1981) concluded that g_j_ in amphibian blastomeres directly depends on pH_i_. In addition, recent studies have shown that pH_i_ modulates Cx36 GJ channel activity through a direct effect on the channel (González-Nieto et al., 2008). In the same study, it was shown that the H18 residue in the NT domain is crucial for the uncoupling effect produced by alkalization. Moreover, others have also reported that low pH_i_ could have a direct effect on Cx hemichannels (Trexler et al., 1999; Sanchez and Verselis, 2014).

Direct interaction of Mg^2+^ ions with Cx36 GJ channels was suggested in Palacios-Prado et al. (2013, 2014). It was proposed that Mg^2+^-dependent gating of Cx36 GJs is a distinct regulatory mechanism, in which the sensitivity of Cx36 GJ channels to high [Mg^2+^]_i_ is determined by aspartate (D47), located in the channel pore (Palacios-Prado et al., 2014).

Our results show that g_j_ of the Cx36 GJ channel during acidification strongly depends on [Mg^2+^]_i_ (Figure 1), but is less affected by alkalization. It is likely that during an intracellular acidification, H^+^ reduces the affinity of Mg^2+^ for its binding site, thus causing an increase of g_j_ at high [Mg^2+^]_i_. This view is supported by the fact that acidic pH_i_ causes the decrease of g_j_ only at low [Mg^2+^]_i_, when the inhibiting effect of Mg^2+^ would be reduced and changes in g_j_ should be mainly determined by H^+^. One possible explanation for such a reduction of affinity could be an interaction between H^+^ and Mg^2+^ for the same negatively charged binding sites. Such an interaction has a strong chemical basis and was previously reported in various studies. For example, Russell and Brodwick (1988) demonstrated that competition of H^+^ and Mg^2+^ ions can affect Cl^−^ fluxes in giant barnacle muscle cells. Mg^2+^ and pH_i_ interaction was also demonstrated for TRPM7 cation channels (Jiang et al., 2005) or P2X7 receptors (Acuña-Castillo et al., 2007). It was proposed that TRPM7 channels have two binding sites for the Mg^2+^ ion, one of which could also be the site for H^+^ binding (Chokshi et al., 2012). Experimental data of this study show that alkalization decreases g_j_ to a similar degree at all levels of [Mg^2+^]_i_, which suggests that the effect of alkalization does not significantly depend on [Mg^2+^]_i_. Moreover, this supports the hypothesis that alkalization of Cx36 has a distinct gating mechanism, as was proposed in González-Nieto et al. (2008). The lack of strong dependence between high pH_i_ and [Mg^2+^]_i_ probably does not require an explicit explanation if one assumes that the competition of H^+^ and Mg^2+^ ions is the driving factor for the combined effect of pH_i_ and [Mg^2+^]_i_. On the other hand, one cannot exclude the possibility that Mg^2+^ ions interact more efficiently with the Cx36 protein under alkaline conditions. Under such a hypothesis, the binding affinity of Mg^2+^ would increase due to reduced [H^+^]_i_, which could stabilize the protein channel in a closed conformation even at low [Mg^2+^]_i_.

The mathematical model used in this study explain the biphasic nature of the g_j_-pH_i_ relationship, assuming the presence of two separate gating mechanisms sensitive to acidic and alkalic conditions, as was proposed by González-Nieto et al. (2008). We presume that the activity of the channel could be modulated by separate sensing domains, which provide different sensitivity through the same gate. Our modelling results (Figure 4) suggest that Mg^2+^ ions might affect both alkalic and acidic gating mechanisms, particularly at low pH_i_.

An indirect effect of alkalization on g_j_ could also be considered. For example, it is known that high pH_i_ increases [Ca^2+^]_i_ via release of Ca^2+^ from the endoplasmic reticulum (Li et al., 2012). However, this is unlikely to be the only mechanism involved, because our results showed no significant difference between g_j_ decreases in control experiments with pipette solutions containing cytosolic Ca^2+^ buffer BAPTA, 10 mM (data not shown). In Palacios-Prado et al. (2013), it was shown that the increase of g_j_ at low [Mg^2+^]_i_ can be seen even in phosphomimetic mutants of Cx36 and using pipette solutions containing BAPTA. This excluded the role of CaMKII kinase, which was previously reported to cause a “run-up” phenomenon in Cx36 GJs (Del Corsso et al., 2012).

### The possible role of NT domain in H^+^ and Mg^2+^ regulation

Mg^2+^ and H^+^ ions have many possible binding sites through which they could affect g_j_. Potential candidates might include various negatively charged residues for divalent cations or histidine residues, which can be protonated at physiological pH_i_ values. For example, computational analysis and crystallography indicate that two glutamates (E42 and E47), residing in the Cx26 GJ channel pore near the extracellular gap could coordinate Ca^2+^ binding (Bennett et al., 2016), which might also be targeted by Mg^2+^. Negatively charged glutamates of the NT domain, which forms the vestibule of the GJ channel (Purnick et al., 2000), could be involved in binding Mg^2+^ ions or facilitate their pass through the channel. At high [Mg^2+^]_i_, E8Q and E12Q mutations abolished the stimulating effect of acidification and double mutation of these two amino acids even enhanced uncoupling in the same conditions. In addition, all mutations increased the uncoupling effect at low [Mg^2+^]_i_. The effect of [Mg^2+^]_i_ on g_j_ at control pH_i_ remained comparable to Cx36-EGFP, with the exception of the E8Q mutation, which eliminated the increase of g_j_ at low [Mg^2+^]_i_ and the most prominent difference was obtained with double mutation, which basically abolished the uncoupling effect of high [Mg^2+^]_i_. Our modelling results indicate that both mutations modified the open channel probability determined by the acidic sensing domain, particularly at high [Mg^2+^]_i_. In particularly, sensitivity of acidic gating to high [Mg^2+^]_i_ is gained in mutants with most prominent manifestation in double mutant (Figures 4E,H,K blue line), while in Cx36 WT no acidic gating sensitivity is observed (Figure 4B blue line). Moreover, double mutation decreased dependence of open channel probability on pH_i_ at different [Mg^2+^]_i_ as compared with Cx36 WT and both single mutations.

The most plausible interpretation of these results might include the direct binding of Mg^2+^ and H^+^ ions to E8 and E12. However, the remaining sensitivity of Cx36^*^E12Q-EGFP to Mg^2+^ ions at control pH_i_ imply that E12 is not a part of the Mg^2+^-binding site. Moreover, the lost sensitivity of Cx36^*^E8Q-EGFP to low [Mg^2+^]_i_ and the remaining response to high [Mg^2+^]_i_ at control pH_i_, suggest that Cx36 could have two or more binding sites with different affinity to Mg^2+^, similar to TRPM7 channels (Chokshi et al., 2012). We could not exclude indirect involvement of E8 and E12 residues in channel modulation by Mg^2+^. These residues are located in the NT domain, which forms the entrance into the channel (Beyer et al., 2012), and their negative charges could favour Mg^2+^ ions to reach the D47 residue, located at the first extracellular loop. Thus, the substitution of E8 or E12 could disturb local [Mg^2+^]_i_ at the channel pore, which might explain why the E8Q mutation causes the loss in sensitivity to high [Mg^2+^]_i_ and the E12Q mutation led to a decrease of g_j_ during acidification at high [Mg^2+^]_i_. The loss of double mutant sensitivity to high [Mg^2+^]_i_ and the increased uncoupling during acidification also imply that these two amino acids are important for Cx36 GJ channels regulation by Mg^2+^ and H^+^.

Overall, the exact mechanism of pH_i_ and [Mg^2+^]_i_ effect on g_j_ and the role of the NT domain are unclear. Further investigations are needed to determine the regulatory sites for acidification and their dependence on Mg^2+^.

### Functional role of [Mg^2+^]_i_, [H^+^]_i_, and their interaction

Our data show that [Mg^2+^]_i_ can modulate the sensitivity of Cx36 channels to pH_i_. The low sensitivity of Cx36 GJs to low pH_i_ was proposed to act as a preventive mechanism of the function of electrical synapses during brain ischemia (González-Nieto et al., 2008). Our results suggest that such mechanism would require normal levels of [Mg^2+^]_i_. There are a number of studies which have demonstrated therapeutic effect of Mg^2+^ in the treatment of brain ischemia (Westermaier et al., 2013), thus it is possible that [Mg^2+^]_i_ and pH_i_ effect on Cx36 plays at least a partial role in the protective mechanisms. For example, the depletion of ATP during brain ischemia (Sato et al., 1984) could induce an increase of [Mg^2+^]_i_ (Henrich and Buckler, 2008), therefore these factors together might coordinate the regulation of electrical coupling. Presumably, under a mild ischemia it might be beneficial to maintain the normal electrical coupling, while the closure of Cx36 GJ channels during a severe ischemia could isolate the damaged regions of cells, thus preventing the further spread of apoptosis.

## Author contributions

LR, VS, and FB: conception of the work, design of experiments, collection, analysis and interpretation of data, drafting of manuscript; TK and NP-P: recorded and analysed the experimental data; MS: constructed and applied mathematical models; NP-P and MS: critically revised the manuscript; VJ: performed experiments.

### Conflict of interest statement

The authors declare that the research was conducted in the absence of any commercial or financial relationships that could be construed as a potential conflict of interest.
